# Design of Photosensitizing Agents for Targeted Antimicrobial Photodynamic Therapy

**DOI:** 10.3390/molecules25225239

**Published:** 2020-11-10

**Authors:** Maxime Klausen, Muhammed Ucuncu, Mark Bradley

**Affiliations:** 1 School of Chemistry and the EPSRC IRC Proteus, University of Edinburgh, Joseph Black Building, David Brewster Road, Edinburgh EH9 3FJ, UK; Mark.Bradley@ed.ac.uk; 2 Department of Analytical Chemistry, Faculty of Pharmacy, Izmir Katip Celebi University, Izmir 35620, Turkey

**Keywords:** photodynamic therapy, photosensitizer, reactive oxygen species, antimicrobial resistance, nanomaterials, antimicrobial peptides

## Abstract

Photodynamic inactivation of microorganisms has gained substantial attention due to its unique mode of action, in which pathogens are unable to generate resistance, and due to the fact that it can be applied in a minimally invasive manner. In photodynamic therapy (PDT), a non-toxic photosensitizer (PS) is activated by a specific wavelength of light and generates highly cytotoxic reactive oxygen species (ROS) such as superoxide (O^2−^, type-I mechanism) or singlet oxygen (^1^O_2_*, type-II mechanism). Although it offers many advantages over conventional treatment methods, ROS-mediated microbial killing is often faced with the issues of accessibility, poor selectivity and off-target damage. Thus, several strategies have been employed to develop target-specific antimicrobial PDT (aPDT). This includes conjugation of known PS building-blocks to either non-specific cationic moieties or target-specific antibiotics and antimicrobial peptides, or combining them with targeting nanomaterials. In this review, we summarise these general strategies and related challenges, and highlight recent developments in targeted aPDT.

## 1. Introduction

The revolutionary discovery and mass production of penicillin in the first half of the 20th century opened a new era in the fight against bacterial infections [1,2], and the development of new antibiotics in the following decades reduced considerably the mortality caused by infectious diseases. Unfortunately, as antibiotics began to be considered as a quick and easy fix to infections, their misuse and abuse have led to the generation of wide-spread antimicrobial resistance (AMR) [3], and, according to a World Health Organization’s (WHO) report [4], the golden era of antibiotics is now coming to an end. Based on the WHO’s estimations, approximately 700,000 deaths are caused by multi-drug resistant (MDR) infections every year. Similar assessments state that this number could reach 10 million by 2050 if no action is taken, which makes research for alternative treatments a vital mission. As bacteria reproduce rapidly and transfer genetic material (often driven through environmental stresses), mutations responsible for resistance mechanisms spread quickly throughout the microbial world and alarming levels of resistance have already been reached. Thus, new therapies immune to resistance are of utmost importance [5,6].

Photodynamic therapy (PDT) utilises a chromophore, typically called a photosensitizer (PS), which is able to “sensitise” the surrounding triplet oxygen upon absorption of light, and produce highly reactive oxygen species (ROS) such as hydroxyl radicals (Type I photo-process) and cytotoxic singlet oxygen (Type II photo-process) [7,8,9,10]. Initially used for the treatment of cancer [11,12,13,14], skin [15] or dental diseases [16], the increase in AMR has drawn the focus of the scientific community to the adaptation of PDT for the treatment of infections [17,18,19,20,21]. Antimicrobial PDT (aPDT) has become a prominent alternative to classic antibiotic treatment thanks to outstanding advantages such as the non-invasive nature of light, ease of application, and above all the absence of possible resistance to ROS in microorganisms [22]. Thus, the application of PDT has extended to virtually all types of pathogens, including bacteria [17,18,19,20,21], fungi [23,24,25], viruses [26,27] and parasites [28,29]. In particular, following the major concerns around MDR bacteria, several new PS have been tested, adapted or developed for aPDT [30]. In this sense, positively charged PS appear promising for aPDT as they can interact electrostatically with the negatively charged bacterial membrane, and the synthesis of several PS functionalised with small cationic functional groups has been reported [31,32,33,34,35]. Nonetheless, two main concerns can arise from this strategy: (i) a poor target selectivity since such cationic species, due to electrostatic interactions with mammalian cells, lead to off-target damage, and (ii) the poor efficiency of aPDT against a certain type of pathogens, in particular Gram− bacteria and biofilms. While Gram+ bacteria are also the cause of serious infectious diseases, more than 90% of Gram− bacteria are considered pathogenic, and since their membrane composition is such that many antibacterial agents simply fail to enter (vide infra), they represent a major threat. Because of these selectivity issues and potential side-effects, and in contrast to the use of PDT in oncology, aPDT has yet to translate widely into clinics [36,37,38,39,40].

In this context, current challenges are focused on an improvement of both the selectivity of PS molecules towards microorganisms, and of their efficiency. Generally, this can be achieved by enhancing the affinity of the PS for specific bacterial components (i.e., membrane proteins etc.), or by disturbing the pathogen to increase its uptake. Practically, known PS building blocks have been co-administered, vectorised or conjugated in a number of different ways so as to incorporate either poly-cationic materials, bacterial-targeting peptides, polymers, antibiotics or antibodies fulfilling either passive or active targeting roles [41,42,43,44]. The synthesis of covalent peptide- or antibiotic-PS conjugates is perhaps the most straightforward way to reach this goal, but supramolecular and nano-approaches have also led to important photo-antimicrobial nanomaterials. In this review, we aim to present the key concepts and underlying challenges in aPDT, with a focus on the most recent work over the past five years in the rapidly expanding field of PS conjugates and PS-containing nanoparticles (NPs). We stress that the focus here is on the photodynamic treatment of infectious diseases, but invite the reader to explore other key applications such as self-disinfecting materials and fabrics [45,46].

## 2. Mechanisms and Challenges in aPDT

### 2.1. Photophysical Principles of PDT

**Figure 1:**
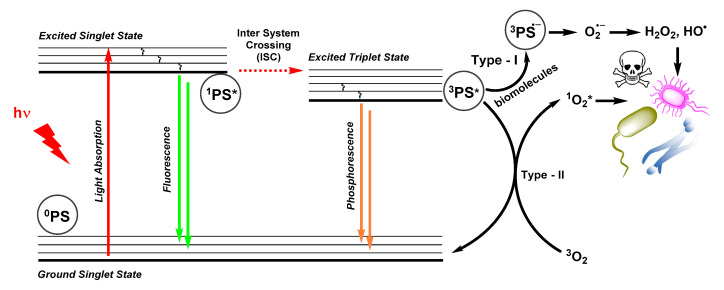
Simplified Jabłoński diagram showing the generation of reactive oxygen species (ROS) by a photosensitizer (PS) following absorption of light, intersystem crossing and Type-I and -II mechanisms. In antimicrobial photodynamic therapy (aPDT), the resulting ROS are able to kill bacteria and fungi as illustrated on the right.

Photodynamic therapy requires three major components: a PS, molecular oxygen (^3^O_2_), and light. The concept is based on the electronic properties of molecular oxygen which naturally resides in a triplet ground state and can therefore interact with triplet-state chromophores. Thus irradiation of a PS by a resonant wavelength of light will excite an electron from ground state ^0^PS to a singlet excited state ^1^PS*, which then undergoes inter-system crossing (ISC) to form a relatively long-lived triplet state ^3^PS*. The efficiency of the ISC process and lifetime of ^3^PS* are two key parameters of a good PS, as this triplet state enables the generation of ROS via Type-I and Type-II photo-processes (Figure 1) [8,10,47]. The Type-I photo-process involves electron transfer between ^3^PS* and a substrate (in a PDT context, generally a biomolecule) to generate a radical anion ^3^PS^.−^ which then interacts with ground-state molecular oxygen (^3^O_2_) to form a superoxide radical (O_2_^.−^). This radical-anion further reacts to generate other ROS following the Haber–Weiss reaction [8,10,47]. In the Type-II mechanism, a triplet-triplet energy transfer occurs between ^3^PS* and ^3^O_2_ to generate singlet molecular oxygen (^1^O_2_*), a short-lived and highly cytotoxic species (energetically the ^3^O_2_ to ^1^O_2_* transition requires 94.3 kJ.mol^−1^). The Type-II photo-process is generally the most common, and its overall photophysical efficiency is expressed in the form of a “singlet oxygen quantum yield” Φ_Δ_, which is a function of the ISC rate constant, the probability of quenching by molecular oxygen, and the efficiency of the triplet-triplet energy transfer. Ultimately, Φ_Δ_ is best expressed as a percentage, and represents the number of ^1^O_2_* molecules generated for 100 photons absorbed.

Thanks to their high “oxidative power”, the ROS generated by the excited PS can be used for the localised killing of cells and microorganisms at the location of the irradiation. Since they are short-lived species, with a lifetime in the microsecond range or below in biological media their “radius of action” varies between a few tens of μm’s in the case of O_2_^.−^ to less than 1 μm for ^1^O_2_*, and even less for the highly reactive hydroxyl radical HO^.^ [10]. To unleash this lethal power on the components of a bacteria, close spatial proximity with their target is vital; which highlights the high potential for localised treatments.

### 2.2. Antimicrobial Mechanisms and Challenges in aPDT

To achieve effective aPDT, the selection of the PS motif is crucial. Although the translation of aPDT into a clinic has not yet fulfilled its potential, decades of reports have provided a series of criteria that need to be fully addressed in the design of a PDT drug.

Suitable optical properties. The PS must be able to absorb light efficiently at the wavelength used for excitation, i.e., with a high extinction coefficient (ε). For a better tissue penetration and lower photo-toxicity near-infrared (NIR) wavelengths of the so-called “photo-therapeutic window” may be preferred [48].

(i) Suitable photosensitizing properties. The triplet state of the PS must possess a long lifetime with efficient ISC, leading to a high singlet oxygen generation quantum yield Φ_Δ_, and/or ROS generation.

(ii) High water solubility. Such compounds, which tend to be aromatic, must be soluble enough to be used in water for biological applications.

(iii) Low dark toxicity. The PS must be non-toxic to mammalian cells prior to irradiation.

(iv) High photo- and storage stability. For application in the clinic the PS must be chemically robust both before and during the irradiation process.

(v) Low long-term sensitization. The PS must be cleared from the body fast enough to prevent long-term sensitization of the patient in daylight. This is particularly important for the treatment of surface infections such as wounds.

(vi) High target selectivity. As mentioned above, the need is for pathogens to be selectively targeted and inactivated without affecting host cells. 

**Figure 2:**
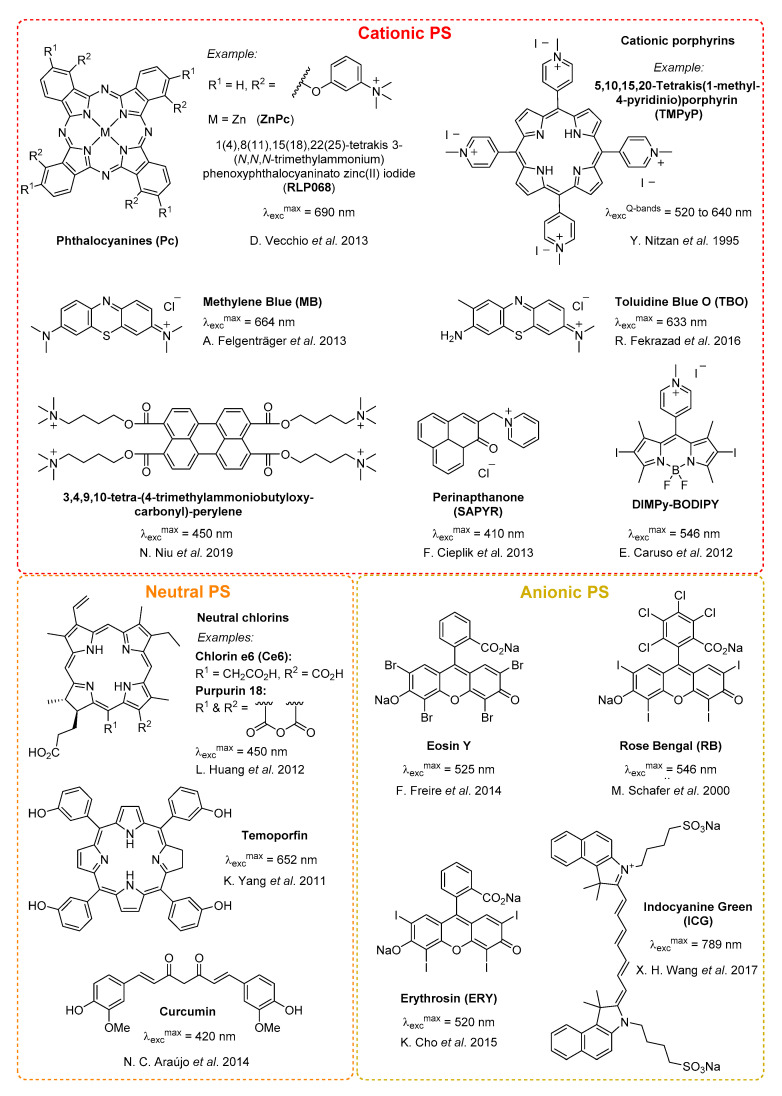
Selected examples of common cationic, neutral and anionic PS used in aPDT.

Thus the properties of many different PDT dyes have been well documented, while many families of compounds have been used to eradicate bacterial species (Figure 2). However, achieving high specificity of treatment while retaining the other criteria is a challenge for aPDT.

Many strategies rely on the electrostatic interaction of the dye with various cell membrane components of the microorganisms (Figure 2). For instance, Gram+ bacteria have a cell membrane composed mainly of a phospholipidic bilayer and peptidoglycans, respectively rich in phosphate and hydroxyl groups, and which, at physiological pH, carry a high density of negative charge, making them susceptible to targeting with cationic PS.

As such it is commonly accepted that cationic PS such as methylene blue (MB) [49], toluidine blue O (TBO) [50] and other phenothiazinium derivatives [51], or cationic porphyrins [52,53] and phthalocyanines (Pc) [54] are well adapted to aPDT, showing both better efficiency and selectivity than neutral [55,56] or negatively-charged sensitisers [57,58,59,60] (Figure 2). Some other synthetic PS decorated with cationic groups to target bacterial infections include BODIPY [31,44,61], perinaphthenone [62] or perylene [35] derivatives. Anionic and neutral PS generally need to be chemically modified or vectorised to eliminate electrostatic repulsion with the bacterial membrane before use in aPDT.

**Figure 3:**
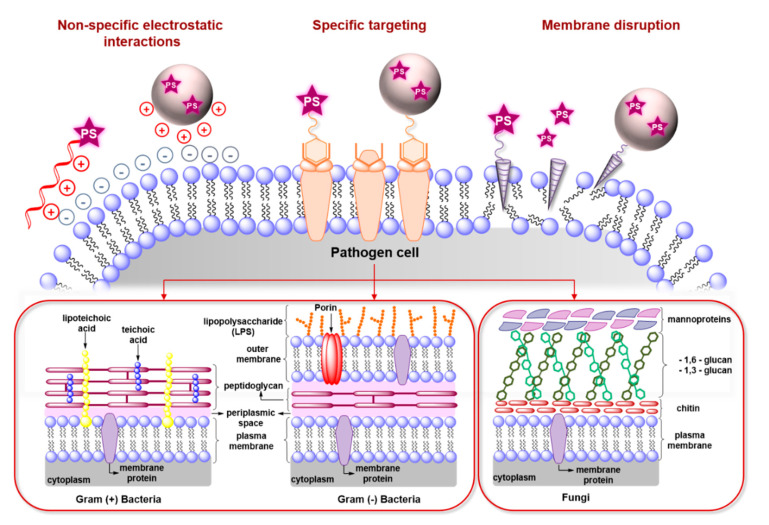
Different strategies applicable in the design of molecular or nano-PS targeted towards pathogens. The different designs involve the conjugation of non-specific poly-cationic materials, specific targeting ligands, or of membrane disrupting agents to known PS, either by direct conjugation to the chromophore, by using nano-carriers, or by co-administration. The lower section shows the differences between the surface of the main types of pathogens targeted by aPDT: Gram+ and Gram− bacteria and fungi.

Light-induced inactivation of pathogens has proven more effective on Gram+ bacteria due to their “single-layered” cell wall/membrane structure that allows deeper penetration of the PS (Figure 3). Thus, whilst aPDT of Gram+ bacteria can be achieved by even mono-cationic PS, poly-cationic derivatives are usually required for the eradication of Gram− bacteria. The presence of a well-organised and thick outer membrane in Gram− bacteria makes them more resistant to aPDT [43] and the thickness of the outer membrane limits the penetration of PS through the cell membrane and wall. This highlights the necessity to design specific sensitisers against such pathogens.

Additional challenges limit the use of small cationic PS as the cell membranes of mammalian cells are also somewhat negatively charged, hence the selectivity towards bacteria is not absolute. In addition, the complex structure of fungal cell walls [63] (Figure 3) also makes them less susceptible to this strategy and more difficult to treat with aPDT. Finally, both types of pathogens are known to generate biofilms in which an extracellular polymer matrix (EPM) protects colonies from outside “aggressions”. Moreover, biofilms are known to be highly pathogenic and hard to treat because of poor drug penetration while displaying a 100- to 1000-fold increase in minimum inhibition concentrations of many drugs [64]. Because of this, more advanced structural modifications are often required, which involves the conjugation of the PS with “passively” targeting poly-cationic or antimicrobial materials, or with “actively” targeting pathogen-specific antimicrobial peptides (AMPs), or antibiotics (Figure 3), as will be discussed in the following sections.

## 3. Recent Studies on Targeted aPDT

### 3.1. Small Molecules & Peptide Conjugates

#### 3.1.1. Conjugation of Small Cationic Groups for Electrostatic Interactions

The development of cationic PS increasing the electrostatic interactions with bacterial surface components (e.g., lipoteichoic acid (LTA) and lipopolysaccharide (LPS) [65]) is a well-known straightforward approach and has been applied for decades [51,53,66]. Porphyrin, Pc, and both their extended and reduced derivatives have been extensively studied and conjugated to different amino-functionalised moieties [53,67]. Building on this approach, a meso-substituted porphyrin derivative bearing four cationic amino groups was developed by Mamone et al. [32] and successfully eradicated Gram+ and Gram− bacteria in both planktonic culture and biofilm models. Interestingly, Li et al. reported a comparative study to investigate the effects of charge on aPDT activity using two poly-cationic zinc-phthalocyanines (ZnPc) conjugated to mono- and di-amino moieties (formal charges of +4, +8) against the Gram− bacteria *E. coli*. As expected, the more highly charged ZnPc derivatives exhibited better inactivation efficacy [33].

**Figure 4:**
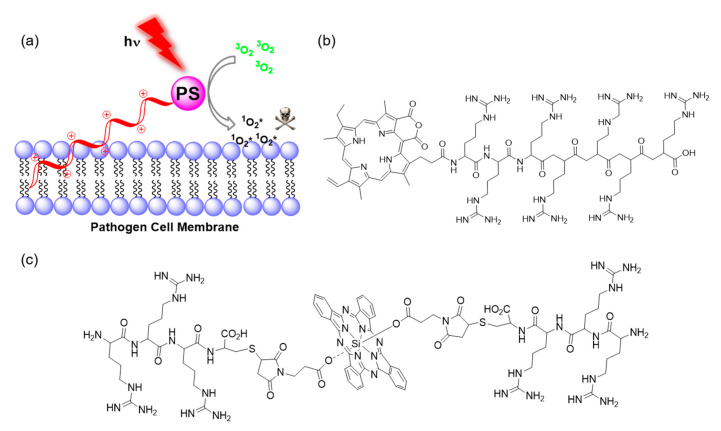
(**a**) General illustration of the interaction between the negatively-charged pathogen cell membranes with poly-cationic materials conjugated to PS building-blocks; (**b**) structure of the hepta-arginine-Purpurin 18 conjugate reported by Zhou et al. [70]; (**c**) structure of the oligo-arginine-SiPC conjugate reported by Zhao et al. [71]. Note: the stereochemistry of the peptides is undefined as it was not specified in the reports.

Basic aminoacid residues (i.e. arginine, lysine and histidine) have the potential to target the negatively charged bacteria surface (Figure 4a). Meng et al. investigated the conjugation of porphyrins to all three basic aminoacids, and investigated their aPDT efficiency [68]. The lysine-porphyrin conjugate was reported to effectively eradicate clinical isolates of bacterial strains including MRSA, *E. coli*, and *P. aeruginosa* both in vivo and in vitro [68,69]. Logically, peptides, containing such building blocks, have been used to take the approach further. Indeed peptide-based strategies offer significant advantages in which the cationic charge can be readily tuned by varying the number of amino acids. The fine-tuning of the hydrophilic/hydrophobic properties and length of the peptide can enhance pathogen selectivity over mammalian cells while enhancing the solubility of aromatic PS dyes. A significant example of this rationale was reported by Zhou et al. [70] with a hepta-arginine peptide functionalised at the *C*-terminus with the hydrophobic purpurin-18 PS (Figure 4b). This probe selectively bound to Gram+ bacteria via electrostatic and hydrophobic interactions, and upon illumination led to complete eradication of Gram+ bacteria. However, the survival rate of Gram− bacteria was high even at high concentrations, presumably due to their thicker cell wall preventing contact of the cell membrane with the PS. In a similar way, Zhao et al. used the membrane binding affinity of arginine-based peptides and developed another probe for aPDT [71] (Figure 4c). They investigated the effect of the number of arginines, substituted axially on a silicon(IV) phthalocyanine (SiPc) PS, on the binding and PDT potency against microbes. Among all the synthesised Arg-SiPc derivatives, the tri-Arg substituted probe showed the highest cellular uptake (strong electrostatic interactions) and phototoxicity against Gram+ and Gram− bacteria as well as fungi in in vitro experiments. In addition, they showed the in vivo therapeutic applicability of this approach with the treatment of *S. aureus* infection in mice models. Importantly, the probe photodynamically inactivated all pathogens with lower IC90 values than the FDA approved photosensitizer MB. Poly-lysine is another well-known type of conjugate in this sense [72], however, poly-cationic PSs can prove toxic to mammalian cells such that the translational potential of such probes can be limited. For this reason, more specific targeting molecules have been used for enhanced uptake and binding to bacteria, with selective agents such as antibiotics.

#### 3.1.2. Antibiotics as Membrane-Disrupting Building-Blocks

In the context of aPDT, the conjugation of well-known antibiotics with photoactive molecules can generate high target selectivity and killing efficacies by targeting and disturbing the membrane integrity of microorganisms. Importantly, this can be achieved with very low concentrations, and without resistance mechanisms being involved since the antibiotic is not directly responsible for the bactericidal activity. In this context, much research has taken advantage of antibiotics to “weaken pathogens” and make them more susceptible to aPDT treatment. The simple co-administration of antibiotics and PDT agents is a straightforward method to seek improvement of the antimicrobial efficacy, however, it is unpredictable and does not always lead to a synergistic effect with higher killing than PDT alone [73,74,75,76,77]. Some recent studies on covalent antibiotic-PS conjugates will be discussed here with respect to their design and mode of action.

**Figure 5:**
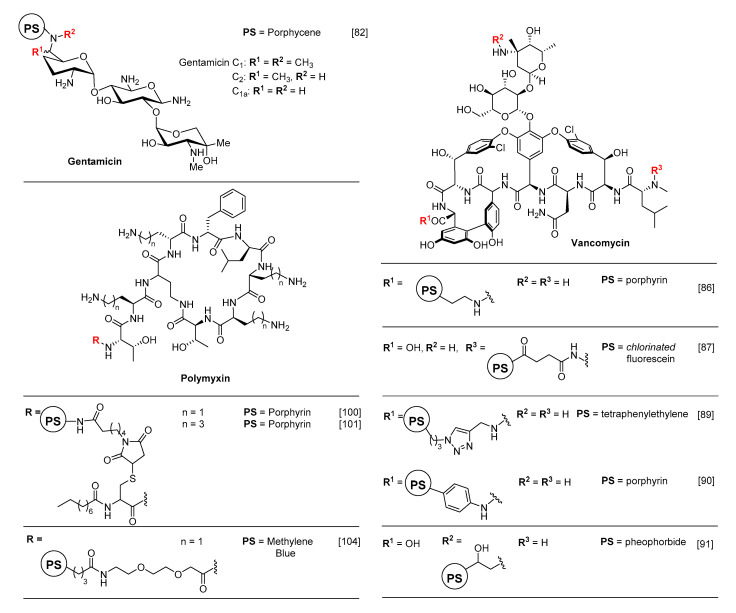
Recent examples of PS-conjugates using antibiotics and antimicrobial peptides (AMPs) as targeting or membrane-disrupting sub-units.

Gentamicin is an aminoglycoside antibiotic first discovered by Weinstein and co-workers in 1963 [78]. It is a mixture of several components and used to treat the broad spectrum of infections caused by Gram− bacteria. Upon contact with the pathogen, gentamicin diffuses through porins in the outer membrane and transfers into the cytosol. The mode of action is based on impedimentation of the initiation and further translation of protein synthesis by binding to 30S and 16S ribosomal RNA [79,80]. The co-administration of this antibiotic with Rose Bengal (RB) in aPDT has been studied by Perez-Laguna et al. [81], and following their work, Nonell and co-workers demonstrated the use of gentamicin as a targeting unit in a covalent conjugation strategy [82] (Figure 5). Their gentamicin aPDT probe, used the red-light-absorbing PS porphycene and showed significant eradication of both Gram+ (*S. aureus*) and Gram− (*E. coli*) strains even at sub-micromolar concentrations while the controls (PS alone and co-administered with gentamicin) did not impact significantly on survival rate. It is important to note that conjugating gentamicin to a PS diminishes its bactericidal activity and attachment of gentamicin to hydrophobic porphycene also enhanced its solubility.

Vancomycin is an antibiotic from the glycopeptide family, used in the treatment of Gram+ infections. Its mechanism relies on the inhibition of cell wall crosslinking by binding to the terminal-D-Ala-D-Ala-OH peptide moieties during the transpeptidation process [83,84]. The conjugation of fluorescent dyes (e.g., IRdye800CW) or PSs to vancomycin to track and eradicate bacterial infections have both been studied extensively [85,86,87,88] (Figure 5). A significant example of this approach was given by Feng et al. in which vancomycin was conjugated to a tetraphenylethene PS with aggregation-induced enhanced emission (AIEE) properties. This theranostic probe allowed selective visualisation and eradication of both vancomycin sensitive and resistant Gram+ bacteria [89]. Zhai and Wang conjugated a tetrakis(*p*-aminophenyl)porphyrin to vancomycin and investigated its aPDT activity against six Gram+ bacteria strains including vancomycin-resistant *E. faecalis* (VRE) [90]. The designed probe showed high selectivity with varying eradication efficacies towards all Gram+ strains. In contrast, a library of photoactive multi-cationic PDT agent was synthesised by Huang et al. [91], and surprisingly, the vancomycin conjugate demonstrated the lowest aPDT activity among all probes. This low efficacy may be the result of a reduced binding affinity of vancomycin due to the conjugation of this multi-cationic bulky PS. Another possible explanation resides in the loss of planarity in the π-conjugated system of the PS sub-unit resulting from its attachment to such a bulky antibiotic, which decreases the extinction coefficient, and therefore the ROS generation efficiency of the probe. Hence, this study reveals the importance of the design of the probe (PS, antibiotic, and spacer), from a chemical, biological and photophysical point of view, to achieve highly efficient aPDT activities.

#### 3.1.3. Antimicrobial Peptide Conjugates

Antimicrobial peptides (AMPs) are linear or cyclic amphipathic peptides that exert a bacteriostatic or bactericidal activity via targeting of the cell membranes of bacteria by electrostatic interactions, and disruption of membrane integrity by insertion, and/or disturbing of intracellular functions [92,93]. More than 2500 AMPs have been reported to date [94] and several of them are used clinically, including for the treatment of MDR infections. As such new targeted aPDT probes have taken advantage of their hybrid interactions, using both electrostatic interactions and membrane anchoring/disrupting capacities, to increase the efficiency of the PS dyes [95]. In a covalent conjugation strategy, decorating AMPs with PS units has attracted substantial attention in the field of aPDT.

Polymyxins (PMX) are non-ribosomal lipopeptide antibiotics of the AMP family used for the treatment of infections caused by Gram− bacteria [96,97]. Their structure is composed of a cyclic hepta-peptide, a tripeptide side chain and hydrophobic tail. Based on the variations of the amino acid sequence at the 6th position on the hepta-peptide, the AMP is denominated as polymyxin-B (D-phenylalanine) or polymyxin-E, also known as colistin (D-leucine). As for many AMPs, the antibacterial mechanism relies on their amphiphilic character, with electrostatic interactions and membrane insertion—leading to entropically enhanced binding. Indeed, the protonated γ-amino units (diaminobutyric acid, Dab) in the cyclic hepta-peptide bind to the outer membrane of bacteria mainly including the negatively charged LPSs. In a second step, the hydrophobic fatty acid tail and D-Phe and D-Leu residues in the cyclic hepta-peptide insert into the outer membrane. This dual mode of action disrupts the cell membrane structure and leads to cell lysis. PMX derivatives are now widely used as last-resort treatment of Gram− infections, and this popularity has led to several examples of conjugation with photoactive molecules (Figure 5).

In this sense, the very first example of aPDT was a synergistic co-administration of PMX with porphyrins reported by Nitzan et al. in 1992 for the photo-inactivation of Gram− bacteria [95]. This non-covalent approach proved that AMPs can be used to increase membrane permeability in the targeted microorganisms and increase PS uptake, a strategy that is still used to this day [98]. In more recent years, Le Guern et al. made substantial efforts to develop photoactive PMX-PS covalent conjugates using the PMX-B scaffold as targeting unit [99,100,101]. A cationic porphyrin was attached to a cysteine-modified PMX-B derivative using thiol-maleimide click chemistry, and the probe exhibited enhanced aPDT efficacy against *S. aureus*, *P. aeruginosa* and *E. coli* compared to the porphyrin alone. Nonetheless, a loss of selectivity was observed, possibly due to the reversible nature of the thiol-maleimide chemistry or the nature of the poly-cationic compounds. In addition, the probe showed dark toxicity with low minimum bactericidal concentrations (MBC) (*P.* *aeruginosa* (10 µM) and *E. coli* (1.2–5.0 µM)), which presumably arises from the bactericidal property of the PMX-B. To diminish this effect, the diaminobutyric acid (Dab) units were replaced with lysines [101], which showed reduced bactericidal activity while maintaining a high aPDT efficacy against both Gram+ and Gram− bacteria.

Building on PMX’s selectivity, Bayat and Karimi generated a targeting tri-branched aPDT probe bearing three colistin (PMX-E) moieties covalently attached to a Zinc phthalocyanine (ZnPc) via random imine formation [102]. To enhance the solubility of this poorly soluble ZnPc-Col probe, it was incorporated, along with glutaraldehyde in different ratios into a chitosan-based hydrogel system (see Section 3.2.2 for more chitosan-based systems). The ZnPc-Col PS embedded into the hydrogels demonstrated variable singlet oxygen efficacies; i.e. the hydrogels with the lowest glutaraldehyde content produced the highest ^1^O_2_* efficiency due to the fast release of ZnPc-Col. This hydrogel strategy allowed the eradication of Gram− *P. aeruginosa* more effectively than the control (ZnPc-glutaraldehyde hydrogel) due to the enhanced solubility of the probe, and increased permeabilisation of the bacteria membrane.

**Figure 6:**
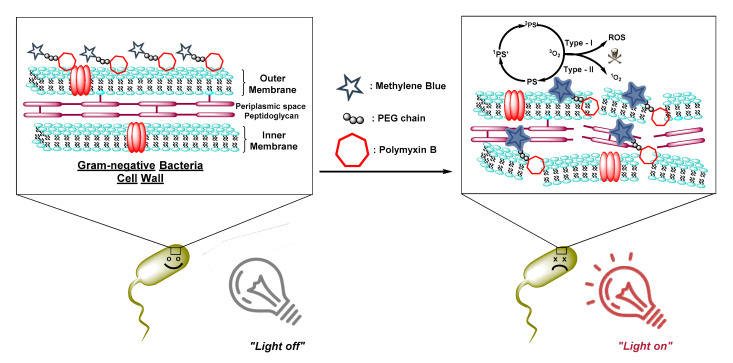
Illustration of the targeted aPDT treatment of Gram− bacteria with the Polymyxins (PMX)- methylene blue (MB) conjugate, during which the PMX cyclopeptide binds to the pathogen membrane and ROS are generated once the light is switched on—an example of entropically driven aPDT.

Following the above studies, we developed an aPDT probe based on the MB and PMX-B sub-units as a part of our ongoing studies on the development of diagnostic and theranostic agents for bacterial infections [103,104]. Most of the above-mentioned aPDT applications were performed against planktonic bacteria. However, the vast majority of infections are associated with biofilms in which the extracellular polymer substances (EPS) provide extra resistance against penetration of antibiotics. In our design, the hydrophobic tail of PMX-B was replaced with a short polyethylene glycol (PEG) linker and attached to a PS (MB) using amine-NHS ester coupling chemistry [104] (Figure 6). In contrast to other studies constructed on the PMX scaffold, the probe exhibited reduced dark toxicity (diminished antibiotic activity) while preserving high Gram− bacteria selectivity (*E. coli* and *P.* *aeruginosa*). In addition to its selectivity, the probe showed outstanding photodynamic bactericidal activity by achieving complete killing of Gram− bacteria in all models including planktonic bacteria, infected skin model, and most importantly biofilms. Furthermore, the absence of detrimental effect on human erythrocytes makes it a significant candidate to treat Gram− bacterial infections without triggering serious side effects.

**Figure 7:**
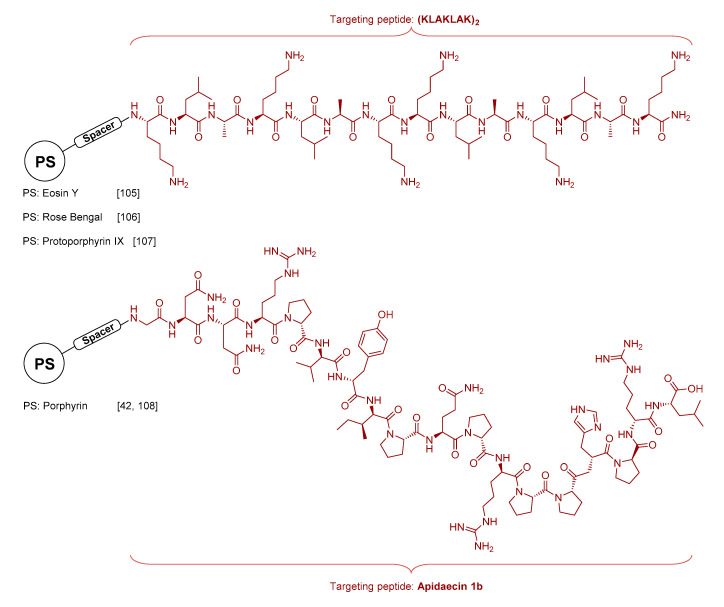
Examples of aPDT probes based on the AMP sequences (KLAKLAK)_2_ and Apidaecin 1b conjugated to different photosensitizing units.

Other significant examples of this approach were reported with the peptide (KLAKLAK)_2_, a prototypical AMP with MIC values in the μM range for *E. coli*, *P. aeruginosa*, and *S. aureus.* Conjugation of this AMP to Eosin Y was successfully reported by Johnson et al. in order to counter the high hydrophilicity and poor membrane affinity of this anionic PS, and actively target Gram+ and Gram− bacteria while retaining good selectivity towards mammalian cells [105]. It is worth mentioning that, recently, Costley et al. reported an RB-AMP conjugate using the same targeting peptide for applications in antibacterial sonodynamic therapy (aSDT) using high-intensity focused ultrasounds as trigger [106]. Cheng et al. also designed a probe composed of this AMP using protoporphyrin IX (PpIX) as a PS [107] (Figure 7). The probe showed excellent photodynamic activity against both Gram+ (*S. aureus*) and Gram− (*E. coli*) bacteria in in vitro experiments. Its high inactivation efficacy relies on both the hydrophobic/hydrophilic structure of peptide and the ROS generation ability of the PS. The AMP unit enables the formation of a α-helical structure that positions the positive charges on one side and leads to strong interactions between the dye and both bacteria surfaces. After initial electrostatic interactions, the AMP-PS conjugates can penetrate into the cell membrane and disrupt cell integrity, while light irradiation at longer wavelength leads to oxidation of biomolecules (e.g., nucleic acids) thus resulting in bacterial killing. As in the above example, the chimeric peptide also eradicated Gram+ bacteria (*S. aureus*) in infected mice models.

Other peptide sequences like Apidaecin 1b [42,108] (Figure 7) or Aurein 1.2 [109] have been used in covalent or non-covalent strategies leading to notable examples of aPDT. In addition to the strategies presented in this section, covalent and non-covalent approaches can also be exploited and complemented with the use of nanomaterials acting as platforms, matrixes or delivery vehicles, thus yielding macro- or nano-photosensitizers with special features for aPDT.

### 3.2. Macro- and Nano-Photosensitizers

In addition to selectivity issues, many PS have poor solubilities and/or have a tendency to self-aggregate in non-photoactive forms, thus impeding ROS or ^1^O_2_* production. Covalent conjugation to peptides and antibiotics can improve this, but this sometimes necessitates long and costly synthesis, without guaranteeing a high efficiency for clinically relevant conditions (i.e., biofilms or MDR strains). In this sense, macro- and nano-PS can offer advantages over free PS, opening access to different types of materials (including biomaterials and biomimetic strategies), techniques, and delivery strategies. Indeed, these structures can act as vehicles with an inherently high PS loading. The local increase in ROS production caused by this high concentration of PDT drugs can improve their killing efficiency, while the incorporation of dyes within a nanoplatform can also increase resistance to photobleaching, and often provide access to an easier functionalisation. This often relies either on supramolecular interactions to construct nano-sized vehicles in which the PDT drug is “encapsulated” [46,110], or on the covalent attachment of the PS on the surface of a nanomaterial [46]. This strategy has been extensively applied in PDT for cancer treatment, but many nano-PS have also been reported over the last two decades for the treatment of infectious diseases. This section will present the different types of macromolecules and nanomedicines used in aPDT over the last 5 years, with a focus on the carriers that passively or actively increase bacterial uptake.

#### 3.2.1. Micelles and Liposomes

Liposomal and micellar formulations are among the most extensively studied nanostructures for biomedical applications. Their structures allow encapsulation of lipophilic drugs in the core of micelles and the membrane of liposomes, or hydrophilic molecules in the core of liposomes. Therefore, such nano-carriers present a high degree of versatility, potentially high PS loading and tuning of the surface properties to enhance bacterial wall targeting and penetration, for instance by shielding the PS and improving the cationic character of the surface layer.

Encapsulating aromatic compounds such as PS into micelles and liposomal structures has been shown to enhance the photophysical properties of the dyes, with singlet oxygen escaping the membranes of the carrier by diffusion [111]. This has been evidenced notably in micellar and liposomal formulations of hematoporphyrins, in which encapsulation of the PS inside the hydrophobic region of the NPs prevented the formation of photo-inactive aggregates [112]. More recently, Sharma et al. also developed an innovative encapsulation system for MB and RB dyes in which the components of the nano-carrier participate in the overall photophysical process [113]. Self-assembly of a copper-based cationic metallo-surfactant and of an anionic surfactant allowed the preparation of either cation-rich or anion-rich vesicles, suitable for encapsulation of anionic RB or cationic MB respectively. In such NPs it was shown that the copper-based surfactant accelerated the ISC process towards the triplet state, which enhanced the singlet oxygen generation of the PS. Efficient killing of MDR *S. aureus* was reported with this nano-carrier, which was also reported as toxic to bacteria in the dark. Liposomes have also been used extensively in the past to improve the efficacy of PS by disrupting the bacterial cell-wall upon contact with the carriers. This strategy has been shown to enhance the uptake of the PS and therefore the killing efficiency, even in the case of resistant strains [114]. In this sense, and because of their strong tendency to self-aggregate which reduces both photophysical efficiency and availability, the encapsulation of porphyrin-type PSs has been extensively studied for the treatment of bacterial [114,115] and fungal infections [116].

**Figure 8:**
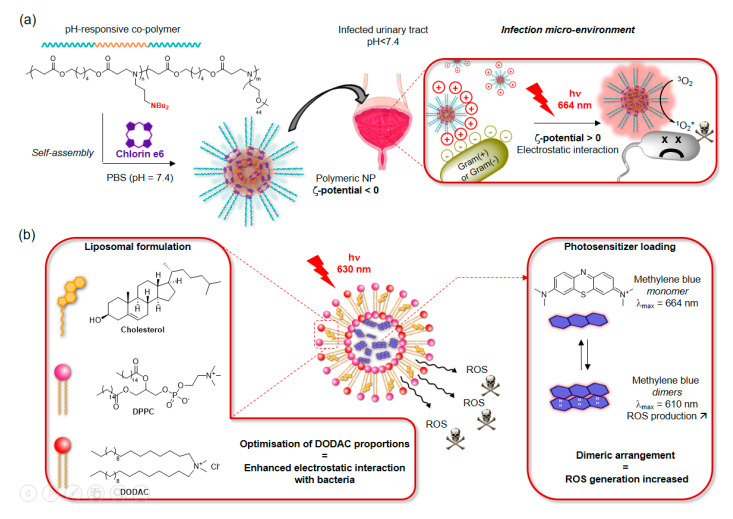
(**a**) Design of Ce6-containing pH-responsive polymeric nanoparticles for the treatment of bacterial infections in slightly acidic media such as the urinary tract [117]. (**b**) Example of liposomal formulation allowing both bacterial targeting via tuning of the surfactant proportions, and of the photosensitisation properties via MB loading [118].

In certain cases, tuning the building-blocks of the carrier can introduce additional targeting properties to the NPs. This can be exemplified in the work by Liu et al. on the encapsulation of Ce6 inside pH-responsive polymeric NPs [117]. The amino-functionalised polymer used for the preparation of the liposomes was designed to be protonated in weakly acidic media such as urinary tract infections environments. As a result, the Ce6-containing polymeric nano-carriers were able to recognise and accumulate on the surface of bacteria via electrostatic interactions at the location of the infection (Figure 8a). This charge-conversion system was able to kill efficiently both Gram− and Gram+ bacteria with MIC values two times lower than the free PS. Cationic liposomes have also been used for the encapsulation of MB, another PS with a tendency to dimerization [118]. A formulation of cholesterol, zwitterionic and cationic lipids gave rise to liposomal structures in which MB would assemble preferentially as a dimer thanks to its high loading, and therefore favour type-I ROS-mediated photo-inactivation of bacteria (Figure 8b). The cationic lipid (DODAC) was employed to test the correlation between the electrostatic interactions with negatively charged bacterial membranes and the overall antibacterial efficacy of the compounds. Importantly, this system showed enhanced penetration in *E. coli* biofilms, and reduced the inflammatory response due to LPS exposure to mammalian cells.

A potential downside of cationic liposomes is their easy fusion with mammalian cell membranes, and the resulting damage. However, the modularity of the self-assembly strategy allows the combination of amphiphilic building blocks with bacterial-specific targeting moieties. Thus, Yang et al. conjugated an AMP (WLBU2) to the surface of temoporfin-loaded liposomes [119]. The low cost and simple applicability of this formulation strategy offer great potential for clinical nano-medical applications. In this sense, it is worth mentioning that the first clinical study using such a nano-medicine was reported by Morgado et al. for the treatment of fungal infections [120]. Their AlPc-loaded nano-emulsion allowed the treatment of onychomycosis without local or systemic side-effects.

#### 3.2.2. Bio-Sourced Oligosaccharide Conjugates

In the world of NPs, bio-sourced macromolecules such as oligosaccharides represent a source of well-documented readily available building blocks for the elaboration of stimulus-responsive nano-systems. Exploiting the properties (charge, supramolecular interactions, functionalisation, etc.) of oligosaccharides has given rise to numerous examples of nano-PS for aPDT. Their preparation relies either on covalent attachment of PS moieties, or encapsulation.

Chitosan is a bio-polymer obtained through deacetylation of chitin. Thanks to its intrinsic poly-cationic nature, it has been reported to interact with the negatively charged phospholipids [121,122], which provides it with a broad-spectrum antibacterial and antifungal action, including the prevention of the development of biofilms [123]. Therefore, poly-cationic chitosan has been one of the most widely utilised delivery and uptake increasing nano-carriers in aPDT.

Early examples of the potentiation of PDT by chitosan goes back to the 2000s, and it has since then been used in co-administration, nano-formulations or covalent attachment of the xanthene derivatives erythrosine [124] and RB [125,126], MB [125,127], Pcs [128,129], or ICG [130] for antibacterial treatments respectively with green to NIR light. Chitosan’s ability to disrupt and permeabilise biofilms is of key importance in improving the efficiency of ROS.

**Figure 9:**
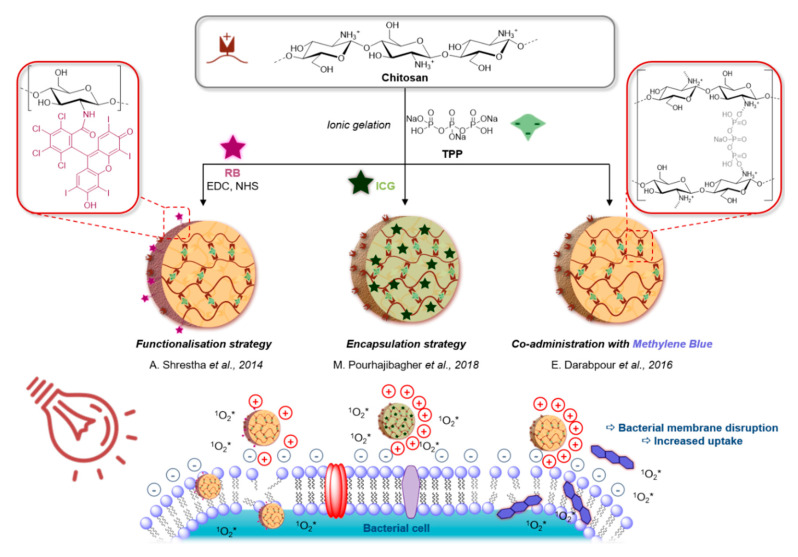
Examples of the preparation of chitosan nanoparticles (NPs) by ionic gelation for aPDT via covalent surface attachment [127], encapsulation [130] or co-adminisation, [126] of different PS.

Among the most recent applications of this strategy, Shrestha et al. tackled the major challenge of dental infections [126]. In an effort to simultaneously eliminate persistent biofilms in root canals, as well as reinforcing the damaged hard architecture of dentin, RB was covalently attached via carbodiimide chemistry to chitosan NPs prepared by ionic gelation method with sodium tripolyphosphate (TPP) (Figure 9). The resulting green-absorbing NPs were successfully demonstrated to be efficient in killing biofilms of *E. faecalis*, where RB alone did not affect the multi-layered biofilm structure. In addition, the photo-activation of the NPs triggered the crosslinking of collagen units in dentin, as well as the incorporation of the chitosan particles within the matrix, which improved its toughness and mechanical properties. Nevertheless, aggregation of the NPs and formation of toxic microparticles are potential limitations of this work. Darabpour et al. later showed that the simple co-administration of chitosan NPs prepared by an ionic gelation method along with 50 µM solutions of MB significantly reduced the viability of *S. aureus*, MRSA, and *P. aeruginosa* biofilms in a synergistic manner upon irradiation [127].

In an alternate strategy, the encapsulation of the PS inside chitosan NPs was reported by Corona and co-workers [129]. Using the NPs as a delivery vehicle allowed them to counter the notorious issues of solubility and aggregation of Pc dyes. Thus, a formulation of Myglyol®, chitosan and chloroaluminium Pc led to positively charged 300 nm NPs that were activatable with 660 nm light, which were effective in the treatment of *S. mutans* biofilms as chlorhexidine digluconates. Interestingly, a series of ZnPc-chitosan covalent conjugates were also prepared for the treatment of fungal infections by Tang et al. [128]. *C albicans* was killed with higher efficiency compared to the PS alone in every case, regardless of the molecular weight of the chitosan derivative used, explained by the reduced tendency to aggregation for the Pc derivative and a higher uptake by the fungi—especially into their mitochondria. Finally, the photodynamic treatment of bacterial biofilms was performed even deeper into the phototherapeutic window in the work of Pourhajibagher et al. with the encapsulation of the NIR absorbing dye ICG inside cationic chitosan [130]. Interestingly, their system resulted in a 91% killing of *A. actinomycetemcomitans* and importantly reduction in biofilms upon irradiation at 810 nm, but was also tested against periodontitis in combination with sonodynamic therapy [130,131].

Cyclodextrins (CDs) are oligosaccharides naturally produced by enzymatic conversion of starch. Their macrocyclic toroidal shape and slightly hydrophobic cavity make them one of the most popular building blocks in supramolecular chemistry and drug delivery. As such, α-, β- and γ-CDs have been used for the preparation of PS-containing NPs, and in recent years porphyrins have been popular dyes for this type of platform, being either covalently [132] or non-covalently bound to CDs [133,134,135,136]. The covalent strategy was used by Ribeiro et al. for the preparation of unsymmetrical Porphyrin-CD conjugates [132], with thiopyridinyl-functionalised PS units cationised in order to improve the solubility of the NPs and their affinity for bacterial membranes. In this study, it was reported that the conjugation to CDs reduced slightly the affinity for Gram− bacteria compared to the PS alone, however the enhanced solubility, availability, and singlet oxygen generation efficiency of the conjugates made them a viable option for aPDT.

**Figure 10:**
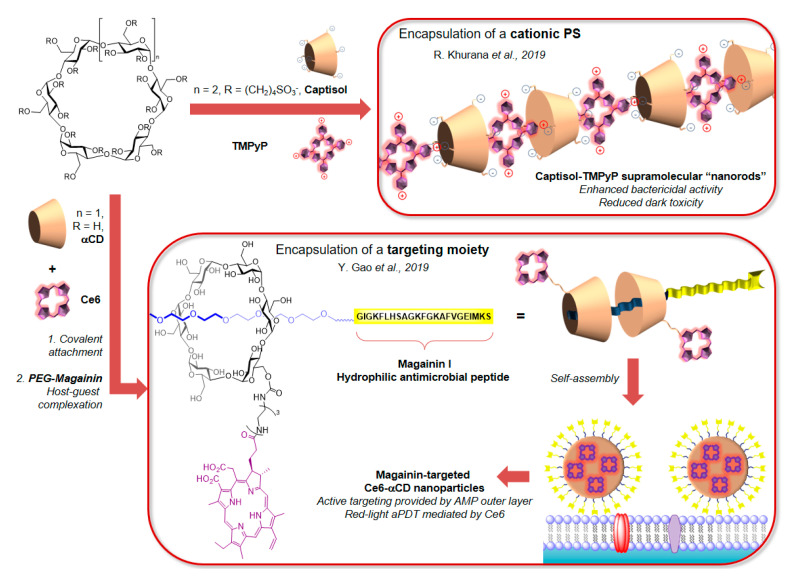
Examples of cyclodextrin derivatives used for the preparation of PS-containing NPs, either by encapsulation of the PS units [136], or by encapsulation of an AMP acting as a bacterial-targeting moiety [137].

A host-guest strategy is often selected for its simplicity, as illustrated by the work of Zagami et al. in which spherical NPs were prepared with quantitative entrapment efficiency by simple mixing of the sulfonate-functionalised β-CD Captisol and the tetracationic TMPyP PS [135]. The same building-blocks were recently applied in the preparation of nanorods by Khurana et al. [136] (Figure 10), which showed decreased dark toxicity of the nano-PS compared to TMPyP. Interestingly, this simple encapsulation strategy has also been used for PS-loaded textiles [134].

Targeting moieties can also be incorporated easily into the supramolecular edifice to improve selectivity. In an elegant supramolecular self-assembly approach, Gao et al. reported an α-CD-Ce6 covalent conjugate in which the CD’s cavity encapsulated a PEG chain terminally functionalised with the antimicrobial peptide Magainin I (M1) [137]. The host-guest complex could be assembled in a micellar structure in which the M1 peptide, facing outwards, acts as the bacterial targeting moiety (Figure 10). This feature allowed a 99.9999% killing efficiency against *P. aeruginosa* and MRSA biofilms using red light. Moreover, thanks to the efficient targeting, the toxicity of the treatment proved less than in the case of the α-CD–Ce6 conjugate alone. It is also worth mentioning that this approach was adapted to fungal infections with the co-encapsulation of the non-water-soluble tetraphenylporphyrin (TPP) PS and the antifungal agent fluconazole in β-CD [138].

Other types of sugar oligomers have been used, including cellulose with PpIX [139], or galactose [140] and maltoheptaose to improve the water solubility of the PS *bis*-iodo-BODIPY [141]. Although the solubility and biocompatibility of the PS are usually enhanced, these oligosaccharides usually do not have an active effect on microbial uptake or targeting, unless they are synthetically modified with cationic groups [139,140] or a bacterial specific moiety [99]. In this sense, it is worth mentioning that Le Guern et al. conjugated both Ce6 and the peptide PMX-B onto the surface of cellulose nanocrystals. The resulting construct was able to eradicate three strains of Gram− bacteria under white light irradiation [99].

#### 3.2.3. Synthetic and other Bio-Inspired Polymer Conjugates

The conjugation of PS units to synthetic macromolecules offers a wide panel of possibilities. Once again, strategies are divided between a straightforward uptake increase triggered by poly-cationic moieties, and the preparation of conjugates with a high level of specificity.

Hyperbranched macromolecules and dendrimers offer interesting structural control options and versatility thanks to the possibility to conjugate or encapsulate. In this sense, Majoral’s phosphorous dendrimers have been adapted to PDT by incorporating known PS units and introducing cationic external layers [142,143]. Although their studies remained fundamental, the authors suggest that MB and RB can be linked to the dendrimers either via electrostatic interactions and π-stacking [142], or by covalent attachment [143]. Interestingly, the latest study revealed that the covalent RB-dendrimer conjugate possessed reduced photo-activity, presumably because of the structural modification of RB, thus impeding its use in aPDT and making the entrapment strategy a more attractive option. In a more applied study, Staegemann et al. prepared hyperbranched polyglycerols co-functionalised with a zinc porphyrin and with mannose units in order to target mannose receptors on the bacterial cell surface [144]. “Click chemistry” allowed covalent attachment of the modules onto the polyglycerol platform, while increased loadings of mannose promoted solubility and bacterial selectivity (*S. aureus*). Unfortunately, the addition of albumin to the culture quenched the aPDT effect which limits clinical application.

**Figure 11:**
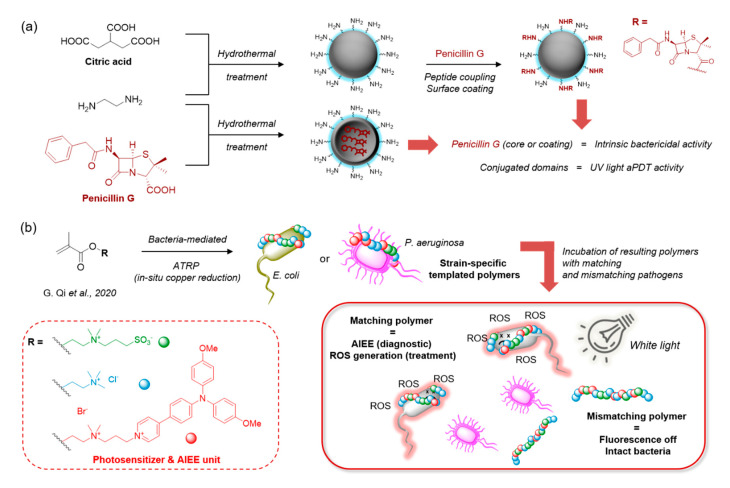
(**a**) Preparation of bactericidal ROS-generating CNPs using Penicillin G as surface targeting moiety or core carbon source [147]. (**b**) Example of controlled co-polymerisation of cationic, zwiterrionic and PS-containing monomers mediated by bacteria, leading to template polymers able to detect and eradicate selectively their original bacterial strain [151].

Going beyond hyperbranched materials, reticulated networks of polymers can lead to carbon nanoparticles (CNPs) or “nanodots” bearing specific chemical and photophysical properties dictated partially by the building-blocks used in the crosslinking reaction. Such materials usually possess intrinsic luminescent properties, and can sometimes be used as PS for aPDT when they present ROS generation properties [145]. In order to create highly cationic CNPs for increased bacterial uptake, Ning et al. performed hydrothermal treatment of a polyfunctional polyethylene-imine in the presence of the dicarboxylate PpIX PS, thus yielding red-absorbing photoactive nano-objects in the 100 nm range [146]. The hydrothermal treatment proved to be a simple procedure requiring no toxic solvents or multi-step synthesis and retained the structure of the PS. However, as is often the case with poly-cationic materials, these CNPs only provided efficient killing of Gram+ bacteria. Targeting capacities could be improved either by surface coating with pathogen-specific units, or by direct modification of the reagents used in the CNP synthesis. Interestingly, these strategies were exemplified by Sidhu et al., who achieved both the coating of standard citric acid-based CNPs with penicillin G, and the direct preparation of penicillin-based CNPs using the antibiotic as a carbon source [147] (Figure 11a), which made the NPs intrinsically hostile towards pathogens with and without light. The use of ampicillin as a carbon source in CNPs was also recently reported, and led to CNPs killing preferentially Gram+ bacteria with ROS generation under visible light irradiation [148]. In addition, by using appropriate building blocks, either in the core or on the surface, the ROS generation efficiency could be fine-tuned by Mandal et al., whose CNPs were derived from anthraquinone derivatives. These could be rendered more photo-toxic by coating the surface of the NP with BSA [149] which results in a reduction of the band gap in the CNPs as evidenced by a red-shifted absorption spectrum, while the creation of more electron–hole pairs leads to increased ROS generation. Moreover, loading these CNP-BSA conjugates with ciprofloxacin led to a dramatic increase in bactericidal efficiency thanks to a synergistic effect between the antibiotic and the ROS.

Bacteria produce a variety of EPS creating a binding network between pathogens. As such, the specificity of this macromolecular substance makes it an inspiration for highly targeted aPDT, and recently, this type of bacterial-targeting biopolymers have been conjugated to appropriate PS. Li et al. thus tagged bacterial exopolysaccharides extracted from *Lactobacillus plantarum* with an anionic RB photosensitizer to prepare bacterial-targeting self-assembling nanoparticles [150]. These NPs possessed an increased singlet oxygen generation capacity compared to RB in solution, improving their killing efficiency for both Gram− and Gram+ bacteria. Qi et al. inspired by the production of EPS, introduced bacteria into an atom transfer radical polymerisation (ATRP) reaction to create templated polymers [151]. The specific sequence of methacrylate monomers in the templated polymer was dictated by their interactions with the bacterial cell wall/membrane, and thus specific to the bacterial strain used. Using a triphenylamine-pyridinium-containing monomer, the templated polymer could not only generate fluorescence by AIEE, but also generate ROS to induce specific bacterial killing under white light irradiation (Figure 11b). This biomimetic strategy could be extended successfully to drug-resistant and clinical strains of Gram− bacteria due to their particular surface components.

#### 3.2.4. Hybrid and Inorganic Nanoparticles

Because of their unique electronic, physical and morphological properties, as well as their possible arrangement into core-shell hybrid structures, inorganic NPs provide boundless versatility and opportunity for biomedical applications. Numerous examples of the conjugation of PS to inorganic or hybrid NPs for aPDT have been reported, however, only a few numbers of them have claimed active pathogen targeting in recent years. Silica and gold NPs are typically employed as nano-carriers, while other materials such as silver and carbon nanotubes have been exploited for their intrinsic antimicrobial properties.

**Figure 12:**
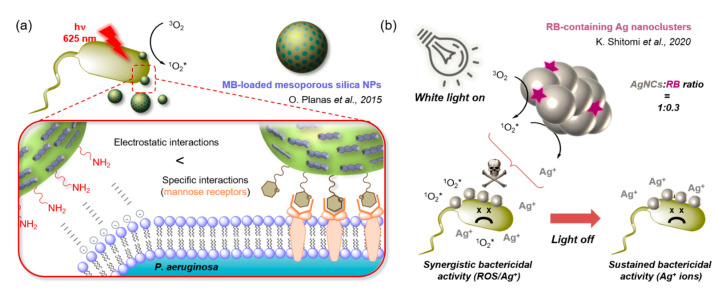
(**a**) Comparison of the bacterial specificity reported for amino- and mannose-functionalised MB-loaded mesoporous silica NPs for aPDT treatment of *P. aeruginosa* [153]; (**b**) Bactericidal modes of action of (RB)-containing silver nano-clusters during and after light irradiation [160].

In the first category, Zhao et al. reported a way to enhance the affinity of Ce6-loaded silica NPs for bacterial membranes by using a poly(allylamine) hydrochloride coating [152]. The design of their nano-system was such that the Ce6 units self-aggregate on the NPs in solution, thus quenching both the emission and singlet oxygen generation properties of the excited state. Upon binding of the cationic coating onto the bacteria, the aggregation state of Ce6 becomes modified, which restores its luminescence and photo-toxicity. This self-activation silica nano-PS allowed complete elimination of MRSA. Nonell and co-workers also reported amino-functionalised mesoporous silica NPs, and co-decorated them with mannose units for additional targeting [153]. MB was adsorbed in the pores of the NPs for aPDT with red-light. In accordance with previous reports, the dark toxicity of the dye was reduced, and in *P. aeruginosa*, the mannose units increased the efficiency of the nano-system compared to the amino-functionalised NPs (Figure 12a). To further potentiate IR-light-mediated aPDT, Grüner et al. designed core–shell NPs also based on mesoporous silica further coated with NaYF4:Yb:Er to exploit energy up-conversion towards the visible [154]. The upconverted energy is then transferred to SiPc loaded in the NPs to generate ^1^O_2_*, while different cationic and anionic coatings allowed comparison of bacterial uptake. As expected, positively charged NPs showed the highest bactericidal efficiency, however, a certain dark toxicity was observed in Gram− bacteria.

Being renowned for its low toxicity, gold has also been used as a platform for targeted aPDT. Exploiting a multifunctional nano-system also used commonly in sensors [155,156], Khan et al. used mannose-based Dextran to cap gold NPs (AuNPs) and aggregate them with Concanavalin-A (ConA), a lectin derivative able to bind mannose and glucose residues in LPS [157]. As a major component of the outer membrane of Gram-negative bacteria, LPS are important markers to consider to increase specificity, and this Dextran-ConA dual-targeting system allowed specific attachment to the fimbriae and to the LPS of bacteria. The resulting AuNPs were loaded with the clinically approved PS MB, which resulted in the effective killing of MDR clinical strains of *E. coli*, *K. pneumoniae* and *E. cloacae*. Interestingly, enhanced singlet oxygen generation was reported in this nano-system thanks to a monomeric arrangement of the MB, which, conversely to the MB liposomes developed by Boccalini et al. [118], favours a type-II photophysical mechanism.

Other types of inorganic nanomaterials have been reported to intrinsically possess certain levels of antimicrobial activity. The bactericidal power of silver has been known since antiquity [158], and recently carbon nanomaterials have also been suggested as potential antibacterial agents [159]. Used as a nano-platform for PS, such materials can give rise to photoactive NPs whose core naturally interacts with pathogens and/or acts in synergy with the photodynamic mediated killing. This synergistic effect has recently been evidenced by Shitomi et al. in RB-containing silver nanoclusters [160]. The ^1^O_2_* generated by RB upon white light irradiation combines with Ag^+^ ions release to kill *S. mutans* in a more efficient way than either alone, while the antibacterial activity of the clusters was reported to be maintained even after irradiation thanks to the released ions (Figure 12,b). Other AgNPs-MB electrostatic conjugates reported by Parasuraman et al. inactivated *P. aeruginosa* and *S. aureus* with enhanced efficiency compared to MB alone, and without significant dark activity [161]. Interestingly, the AgNPs were prepared by bacteria-mediated synthesis, and the biogenic AgNPs enhanced the uptake of MB by pathogenic strains.

A similar strategy was applied with single- and multi-wall carbon nanotubes (SWCNTs and MWCNTs) due to their potent interactions with bacteria and their large surface areas. In one approach, Sah et al. used carboxylic acid-modified SWCNTs as a platform for attachment of amino functionalised tetraphenylporphyrin (TPP) photosensitizers [162] and used this system against *S. aureus*. Non-covalent encapsulation of TBO and MB dyes in MWCNTs was reported by Busi and co-workers [163,164]. Carbon nanographene oxide is another typical carbon material that has been applied in this strategy, and was used as a vehicle for NIR absorbing ICG [165]. The latest system proved to be 1.3 times more efficient than ICG alone against *E. faecalis* in the treatment of endodontic infections. The drawbacks of this strategy reside in the absence of microbial targeting and potential dark toxicity or side-effects.

Remarkably, coated multifunctional NPs exploiting the properties of different materials can also be prepared. As such, a theranostic nano-system integrating both surface-enhanced Raman scattering (SERS) and aPDT was elaborated by Zhou et al. [166]. Silver-coated AuNPs served as a SERS-active core material for diagnostic purposes, and were further encapsulated in silica. Further conjugation of this material with a NIR-absorbing naphthalocyanine PS, and covalent attachment of vancomycin enabled highly efficient imaging and treatment of vancomycin-resistant bacterial strains, including in mice models. Finally, vancomycin was given a dual role in a slightly different approach by Zou et al. [167]. The reducing properties of vancomycin allowed the one-pot preparation of CuS nanocomposites in which the antibiotic was also used as a Gram+ targeting agent. The strong NIR photo-activity of the copper core allows synergistic photodynamic and photothermal killing of resistant Gram+ bacteria. This novel antibacterial photo-material was also tested with success against vancomycin-resistant strains of bacteria in mice infection models.

#### 3.2.5. Immunoconjugates and Protein Conjugates

High molecular weight bio-macromolecules such as antibodies or proteins can sometimes be used as a platform for PDT drugs, and potentially increase the level of specificity. Immunoconjugates prepared by the combination of specific antibodies with drugs have been used in aPDT since the early 1990s [168,169,170]. As such, Protein A, expressed in the bacterial cell wall of *S. aureus* has been a target of choice in this field, opening up new ways for the treatment of MRSA infections [41,170]. The selective lethal photosensitization of *S. aureus* [170] and MRSA [41] was reported by conjugating chlorins and bacteriochlorins to immunoglobulin G (IgG). An alternative targeting strategy was reported by Suci et al. [171]. Their research focused on a viral protein cage modified at reactive cysteines as an attachment point for a ruthenium-based PS. The resulting NPs were further modified with poly-lysine for non-specific bacterial targeting, and with a monoclonal antibody specific to protein A. With this nano-platform, the authors claimed that the delivery of PS per binding event was 45 times higher than in the case of a PS-IgG immunoconjugate. A Ce6 immunoconjugate was also used for whole blood bacterial inactivation by Kim et al. [172]. The red-absorbing PS was conjugated to a polyclonal antibody to *S. aureus* and to a penicillin-binding protein 2a monoclonal antibody for MRSA treatment. The compounds were photo-activated within a thin transparent tube allowing ample and efficient illumination with 83 to 99.9% of bacteria successfully killed without significant off-target damage or changes in red blood cell number. The main drawback of the immunoconjugate strategy is usually the cost of the targeting ligands, which will render the treatment expensive and less clinically relevant in developing countries.

Regarding protein conjugates, Cantelli et al. recently used carbodiimide chemistry to randomly conjugate RB to the homo-tetrameric lectin ConA [173] with an average addition of 2.4 PS per targeting ligand, and a 10-fold higher superoxide generation compared to the model compound. With improved recognition of LPS in the Gram− bacterial membrane, it showed a 4.5-fold higher uptake and greater photodynamic effect. The enhanced targeting of Gram− bacteria by this conjugate improved, up to 117-fold, the bacterial killing of planktonic *E. coli* compared to RB alone. The self-assembly of a tetraphenylethylene-based organoplatinum(II) metallacycle with the coat protein of tobacco virus, conjugated by click chemistry to a transacting activator of transduction (TAT) peptide, was also reported by Gao et al. [174]. The TAT peptide drives internalisation, enhancing the aPDT effect against Gram− bacteria in particular, while the use of this platinum-based PS provided both AIEE properties and enhanced ROS generation via the heavy atom effect. It is also interesting to note that some proteins exhibit naturally ROS generation properties, which opens new treatment perspectives for the future [175,176].

## 4. Conclusions and Perspectives

Because no resistance mechanism to singlet oxygen or ROS has been reported, nor would be expected to arise due to their multi-faceted and generic killing mechanisms, targeted aPDT offers a novel and viable alternative in an era where antibiotics may no longer be long-term options. Nonetheless, translating the potential of aPDT dyes into patients requires considerable efforts, in particular, it is crucial to diminish off-target damage to healthy cells.

The correct selection of a targeting moiety to combine with an aPDT dye is the first step in this direction. As presented in this review, a wide panel of covalent, non-covalent, supramolecular and nano-based strategies are available in the scientists’ toolbox to reach this goal, and the examples reported over the past half-decade hold great promise for the future. However, in spite of its great potential, the widespread use of aPDT in clinics could be hampered by the lack of point-of-care devices for irradiation and treatment of localised infections. Thus the development of clinically relevant user-friendly light-emitting devices [177,178], such as portable light sources or optical fibers, is also of utmost importance in order to adapt the treatment to both superficial and internal infections. Therefore, transdisciplinary research is the key to future breakthroughs in the field.
